# A revised digital edition of Wurm & Hattori’s Language Atlas of the Pacific Area

**DOI:** 10.1038/s41597-024-03816-w

**Published:** 2024-08-29

**Authors:** Robert Forkel, Harald Hammarström

**Affiliations:** 1 Max Planck Institute for Evolutionary Anthropology, Department of Linguistic and Cultural Evolution, Leipzig, 04103 Germany; 2 Uppsala University, Department of Linguistics and Philology, Uppsala, 751 26 Sweden

**Keywords:** Geography, Environmental social sciences

## Abstract

Wurm & Hattori’s *Language Atlas of the Pacific Area* describes the geographic speaker areas of the languages and language varieties spoken in the Pacific. Thanks to the efforts of the Electronic Cultural Atlas Initiative, this monumental piece of work has been available in digital form for over 15 years. But lacking proper identification of language varieties, this digitized data was largely unusable for today’s research methods. We turned ECAI’s digitized artefacts of the Language Atlas into an open, reusable geo-referenced dataset of speaker area polygons for a quarter of the world’s languages. This allows for much more refined analysis methods to, for example, analyse language contact in the area of the world with the highest linguistic diversity. We also describe a number of tool applications and quality checks which may be useful for methodological development in similar digitization efforts.

## Background & Summary

There are some 7,600 languages spoken in the world. Studying these languages – even just cataloging them – typically requires making reference to where they are spoken^1–3^. These references range from coarse grained *macro-areas*^4^ over lists of countries to point coordinates, specifying some sort of center point for the area a language is spoken in. With Glottolog^2^, center-point coordinates are freely available for all languages. While the coarse-grained references largely serve as organization device, for example to stratify language samples, point coordinates have been used to relate languages to environmental data in numerous studies^5,6^. But while useful to some extent, center-point coordinates – even when extended to polygons using algorithmic procedures like Voronoi tessellation^7,8^ – are insufficient for many research questions in historical or diversity linguistics, for example when “environmental conditions present in the territory of a population speaking a specific language”^9^ are of interest.

Thus, an open dataset showing the geographic extensions of languages is highly desirable^10–13^. As Rantanen *et al*.^13^ put it:

“Despite remarkable progress in digital linguistics, extensive databases of geographical language distributions are missing. This hampers both studies on language spatiality and public outreach of language diversity.”

With the dataset described in this paper^14^, we contribute a set of speaker area polygons for languages mapped in Wurm & Hattori’s seminal *Language Atlas of the Pacific Area* (in the following just *the Atlas*). It was originally published in two instalments 1981 and 1983^15,16^ constituting 47 printed coloured maps (“leaves”) and accompanying language lists. The dataset covers a total of 1,769 different languages from 90 different families (by the language classification of Glottolog) — about a quarter of today’s roughly 7,600 spoken languages. The dataset includes virtually all Austronesian languages, thus covers the second largest language family of the world. In addition, all of New Guinea — arguably the place on earth with the highest linguistic diversity — is covered entirely. Apart from its breadth, the Atlas is renowned for its quality^17^^,^^11-12^. Although our understanding of the language inventory and geography is continually improving, the Atlas is remarkably durable and is a valid resource even with today’s knowledge.

**Fig. 1:**
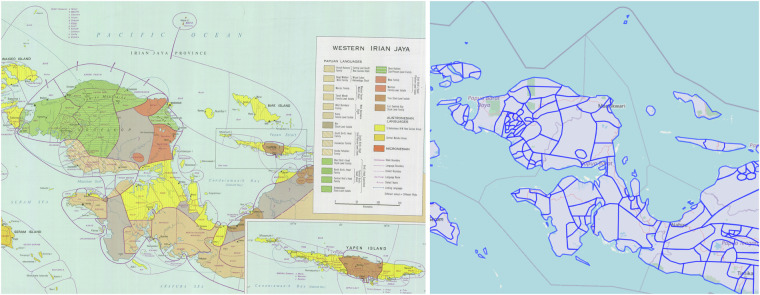
This figure illustrates the starting point for this revised edition of a dataset based on Wurm & Hattori: A scan of a leaf from the Language Atlas of the Pacific Area and the shapefile, digitized by ECAI, overlaid over a base map compiled from OpenStreetMap data.

The starting point for our dataset are the results of the digitization effort by the Pacific Language Mapping project (https://ecai.org//austronesiaweb/pacificlanguages.htm) of the Electronic Cultural Atlas Initiative (ECAI) (see Fig. 1). Considering the often argued need for “language polygon” datasets^10,13^ it seems puzzling that the ECAI data has not seen much use in linguistics. But further investigation revealed that an essential ingredient was missing to make the data readily usable. ECAI laid the groundwork by geo-referencing the Atlas leaves and “transcribing” the depicted areas into polygons in a GIS system, but the resulting shapefile (in the following just *the shapefile*) does not link these polygons to languages using any kind of language identifier. Linking the roughly 4,500 shapes based on the (minimal) metadata they provide to the roughly 2,000 languages spoken in the region is the non-trivial task making up the core of our contribution. We are definitely “standing on the shoulders of giants” here, but at the same time we take the essential step to help Wurm and Hattori’s Atlas as well as ECAI’s digitization fulfill its true potential.

The shapes describing speaker areas from the ECAI dataset are clearly superior to centroid coordinates for languages, e.g. because they can convey the information that languages are spoken in multiple, separate areas – as are many Polynesian languages. They are also more informative than algorithmic approximations of speaker areas, such as a Voronoi tessellation, because they can depict situations where a language is completely surrounded by another language, or can leave out uninhabited areas. Still, the shapes in this dataset are an abstraction, only a proxy for “true” speaker areas. For example for many small islands and reefs in the Pacific, the shapes do not reflect true land areas, but are often larger triangles – in order to make tiny land masses visible at all. Thus, as with other datasets of speaker areas for languages, the caveat formulated by Bowern 2021^18^ applies:

“These maps should be considered as a way to relate linguistic groups to one another in space. It should help to visualize the approximate distances between groups, abstracting away from multilingualism, population density, and Indigenous settlement patterns.”

## Methods

The dataset presented here was derived from the data released by ECAI’s Pacific Language Mapping project (https://ecai.org//austronesiaweb/pacificlanguages.htm). In addition to scanning the leaves of the original Atlas^19^, a “geo-registered GIS dataset”^20^ was created and made available at https://ecaidata.org/organization/ecai-pacific-language-mapping. Fortunately, the data was released under licenses allowing for re-use of the data including creating derived works.

Scans of the five leaves covering Japan have not been made available by ECAI and Japan is consequently not covered in the shapefile. This limitation can be considered minor, though, because we are interested in creating a polygon dataset on the language level and the Atlas leaves on Japan cover at most a dozen languages and deal mostly with dialect variation.

Artefacts created in the data curation process are available in a repository on GitHub (https://github.com/cldf-datasets/languageatlasofthepacificarea).

### Geo-referencing the Atlas leaves

A subset of ECAI’s scans – the 12 leaves covering New Guinea – have been released as geo-referenced JPEG images; the image file is accompanied by a so-called “world file”, describing an affine transformation which will bring the map depicted in the image in agreement with a map projection or Coordinate Reference System (CRS). No information is given, though, on the target projection. Thus, we assume the CRS to be EPSG:4326 – which is the default in common GIS software. Since we wanted geo-referenced images for all Atlas leaves and preferably in EPSG:3857 (“web mercator” projection) in order to facilitate quality control later on, we decided to geo-reference from scratch, starting out with the scans provided by ECAI. In order to increase interoperability considering the issues related to the antimeridian (see below), we cut ECAI’s leaf L019 at longitude 180° into two leaves – L019a and L019b – and geo-referenced both separately.

**Fig. 2:**
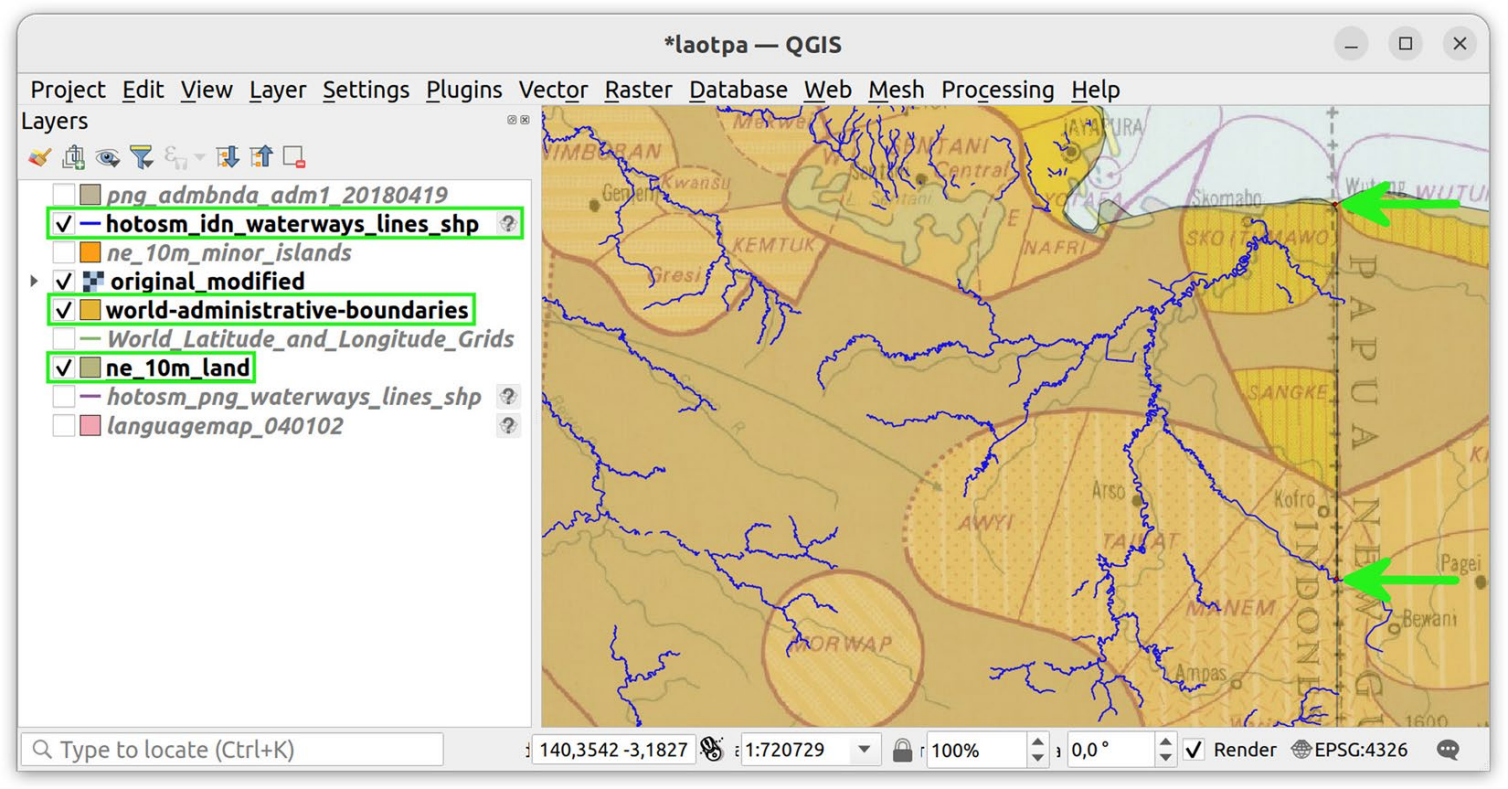
Geo-referencing Atlas leaves with QGIS: The screenshot shows a selection of vector data layers overlaid on an Atlas leaf. The top arrow shows a control point obtained by matching the intersection of country border and coastline in the Atlas leaf with the corresponding point from the administrative boundaries and coastline vector layers. The bottom arrow points at one defined by the intersection of a country border and a major waterway.

Geo-referencing was carried out using the QGIS software^21^, setting ground control points based on coastline features, administrative boundaries or waterways, which are all available as shapefiles for CRS EPSG:4326 (see Fig. 2). Since we know nothing about the projection used in the original Atlas (and the lack of meridians and parallels in the printed maps making any guessing difficult) and not much about how ECAI’s geo-referencing affected the scans, we tried to use many control points and then work the list of available transformation algorithms (https://docs.qgis.org/3.34/en/docs/user_manual/working_with_raster/georeferencer.html) from simplest (“linear”) to most complex (“thin plate spine”) accepting the results of the first one providing a “good enough” fit, judged by inspection. This step resulted in a geo-referenced image for the projected CRS EPSG:4326 in GeoTIFF format for each Atlas leave.

To make it possible to use the geo-referenced images as overlay in web maps, they must be re-projected to EPSG:3857 and translated to an image format that browsers can handle. For the first step, we used the rasterio package^22^, for the second step we used the gdal_translate command from the GDAL software library^23^. This re-projection and image creation process is implemented as the webmercator command in our Python package cldfgeojson^24^. To make the resulting JPEG images accessible for libraries like leaflet, which cannot handle GeoTIFF, we recorded the geographic bounding box of the GeoTIFF files, i.e. the output of the rio bounds command run on the GeoTIFF files.

### “Language-referencing” the shapefile contents

The GIS dataset from ECAI is made available as an ESRI shapefile. It contains 4,483 shapes, 4,467 of type *Polygon* and 16 of type *MultiPolygon* (when translated to geometry types of the GeoJSON specification^25^), described with a small set of metadata. While a MultiPolygon type was sometimes used, it is still often the case that multiple individual shapes of type Polygon describe the same linguistic entity based on identical sets of metadata. For example, there are 39 Polygon shapes covering the islands in Polynesia where Tuamotuan is spoken, providing a language specification of PA’UMOTU as single piece of metadata.

As a first step towards “language-referencing” the shapes, we normalized the metadata, fixing typos in field names etc. The implementation of this normalization is available at https://github.com/cldf-datasets/languageatlasofthepacificarea/blob/main/lib/metadata.py). After normalization, we ended up with four metadata fields: “LANGUAGE”, “COUNTRY_NAME”, “ISLAND_NAME” and “SOVEREIGN”. The most important piece of information is clearly the specification of a language variety provided in the “LANGUAGE” field. These specifications were copied from labels in the Atlas. The other three fields are populated less consistently and provide information useful for disambiguation in case of identical language names. The least frequent field “SOVEREIGN” provides information for cases like Christmas Island where “Christmas Island” is the value for the “COUNTRY_NAME” field and “Australia” is given as “SOVEREIGN”. During normalization we could also exclude polygons marked as not carrying information about a linguistic entity, for example shapes characterized as describing uninhabited areas via a “LANGUAGE” value of “Uninhabited”.

This resulted in 3,074 unique sets of values for the four remaining metadata fields describing 4,294 of the 4,483 shapes. Assuming that linguistic entities are uniquely characterized by such a metadata set, these 3,074 items constituted our to-do list for the “language-referencing” step. The table in the GitHub repository at https://github.com/cldf-datasets/languageatlasofthepacificarea/blob/main/etc/languages.csv includes these 3,074 metadata sets, adding some more that were introduced by fixing incorrect or incomplete metadata as specified in the table at https://github.com/cldf-datasets/languageatlasofthepacificarea/blob/main/etc/fixes_metadata.csv.

We match shapes (identified by metadata sets) to the identifiers of the Glottolog catalogue^2^ which in turn can be used to find other metadata, references, classification and so on of the language in question. This catalogue has identifiers^26^ for three levels: dialect, language and (sub-)family (with *languoid* as the level-neutral term). The majority of matches are at the language-level but the other levels provided flexibility to either be more specific – in case a matching Glottolog dialect was identified – or at least assign a Glottolog sub-family, based on classificatory information in the Atlas. Glottolog’s language-level identifiers are nearly one-to-one interchangeable with other language inventory codes such as ISO 639-3 (https://iso639-3.sil.org/)^26^.

Matching a metadata set to a Glottolog languoid^27^ was done in a multi-step, iterative process. A first pass was done automatically (and only slightly geographically informed via the “COUNTRY_NAME” property), matching based on identical language names and country information. Note that this introduced some false positives due to the multitude of alternative names used to designate languages. Thus, in a second step, the matches were checked geographically by testing whether the polygons assigned to a Glottolog language actually contained the center point for this language given in Glottolog. This process resulted in some matches being rejected and some Glottolog center-points being corrected.

**Fig. 3:**
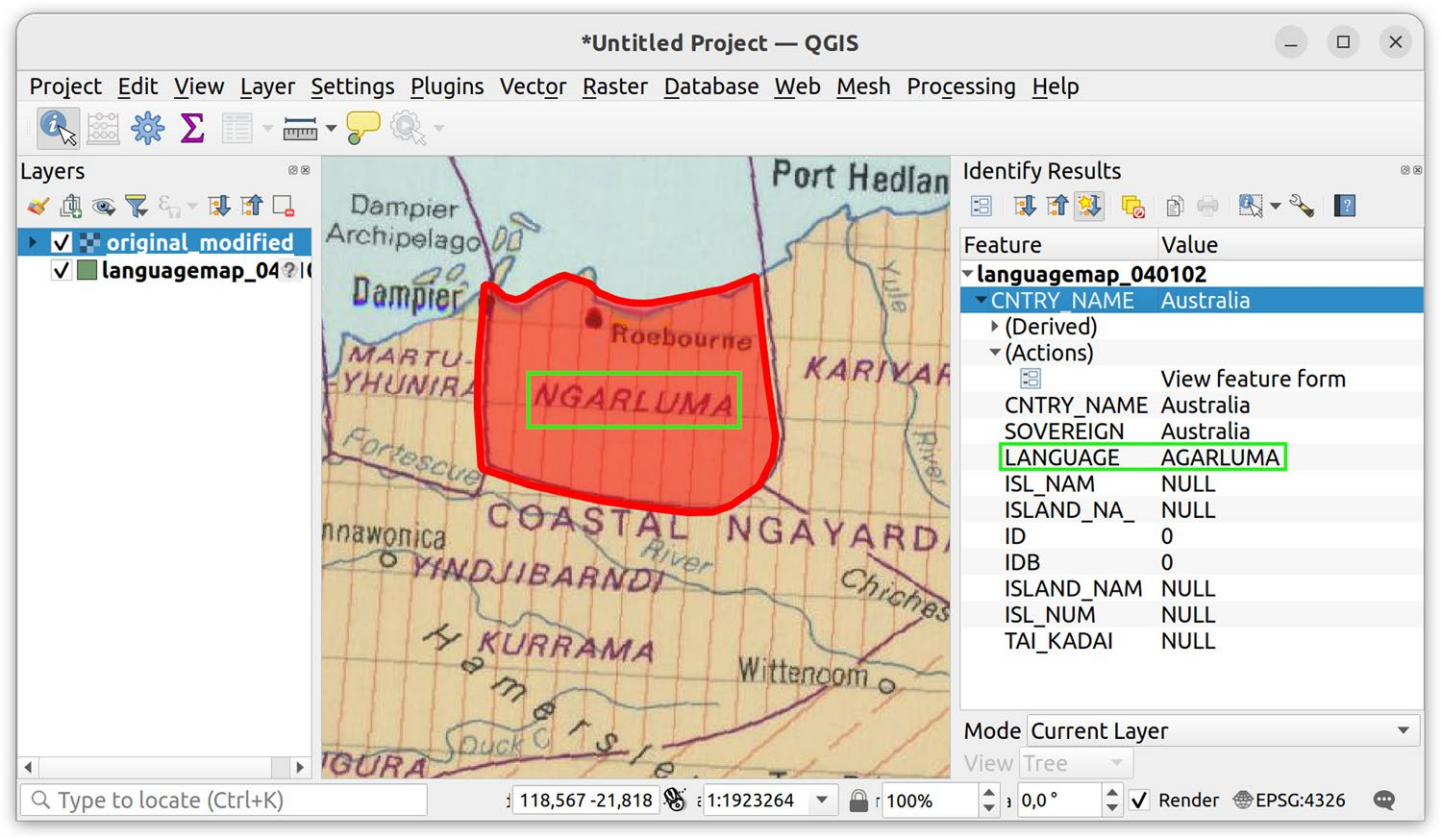
Incorrect transcriptions of Atlas labels could be detected by overlaying geo-referenced Atlas leaves with the shapefile content using the QGIS software. The figure shows the shape with LANGUAGE property AGARLUMA matching the area of the geo-referenced Atlas leaf labeled NGARLUMA.

After bootstrapping the referencing process using automated methods, the next step was done using expert judgements. To provide the full extent of information available in the Atlas when assessing a polygon, we overlaid the polygons over the geo-referenced Atlas leaves. Having information about neighboring languages and dialects available as well as the classification information given in the Atlas proved key to solving many cases — and was essential to solve issues such as typos that happened when Atlas data was transcribed into shape metadata (see Fig. 3). Inspecting the polygon data overlaid on the Atlas leaves revealed another class of issues that was harder to address: Some polygons were only labeled with the classification information despite the Atlas having explicit language names. We added all such cases to an “errata list” (https://github.com/cldf-datasets/languageatlasofthepacificarea/blob/main/etc/fixes_metadata.csv) which also describes a remedy by specifying the appropriate metadata values. So, when encountering a language label from our errata list, we check whether any of the points listed in the errata falls within the associated polygon, and if so, adjust the polygon’s metadata. Once this is done, the “normal” language-referencing via our (extended) to-do list can proceed.

In each individual case the following information was available for assessing matches to Glottolog:

- A language (or dialect) name given in the shape metadata (languages being spelled in uppercase, dialects in mixed case)
- A language (and often a dialect) name in the Atlas
- Classification information given in the Atlas (typically by giving a “stock” and “phylum” and sometimes “family” name)

In the case of New Guinea, we also could make use of Lois Carrington’s bibliography^28^, giving alternative names and describing language and dialect relations, explicitly listing names from the Atlas.

This information needed to be matched against the following information in Glottolog:

- A languoid name
- Full genealogical classification information
- A geographic point coordinate
- Alternative names aggregated from several other language catalogs such as Ruhlen^29^, WALS^30^, ElCat^3^, AustLang^31^ or MultiTree^32^

Particular care was taken to match names that only differed in capitalization. While often one dialect of a language has the same name as the language, there are cases where the dialect name denotes a dialect of a different language (for example *Wambon* denotes a dialect of *KAETI* not of the neighbouring language *WAMBON*, see^33^).

Of the 3,086 metadata sets of the extended language list, all but 25 could be matched to a Glottolog languoid, 2,097 to a language, 803 to a dialect and 165 to a (sub-)family. In 4 cases, polygons were determined as marking overlapping areas in the Atlas, and thus matched to two languoids. The result of this matching process was saved in tabular form (https://github.com/cldf-datasets/languageatlasofthepacificarea/blob/main/etc/languages.csv) and used to inform the creation of the final dataset.

### Correcting polygons

#### Invalid geometries

**Fig. 4:**
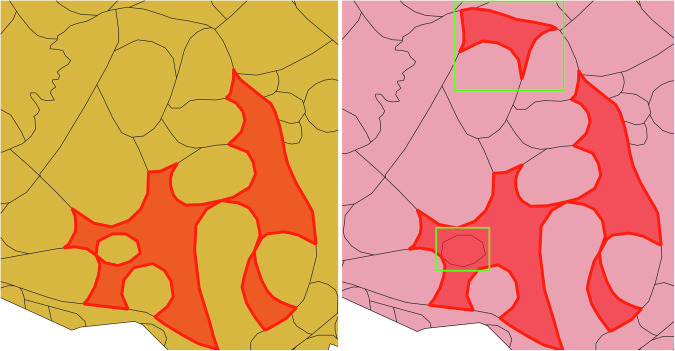
This figure shows the effect of (i) aggregating polygons with matching metadata into MultiPolygon shapes and (ii) fixing invalid geometries. The shape on the left (from the shapefile) was drawn as single, self-intersecting ring and was turned into a proper polygon (on the right) by replacing it with its hull, removing the not explicitly marked hole. The highlighted polygon on the top right side was added to the shape due to identical metadata.

ISO 19125-1:2004 Geographic information - Simple feature access^34^ defines a polygon as a list of non-intersecting rings such that the first ring specifies the exterior ring of the polygon and (optional) additional rings specify holes in the surface bounded by the exterior ring. With “hand-drawn” polygons from GIS systems it sometimes happens that the resulting geometries do not conform to this specification, e.g. because polygons contain self-intersecting rings. In the shapefile we encountered two such cases and fixed them by replacing the self-intersecting ring with the first ring of the zero-distance buffer around the geometry (see Fig. 4). This fix can be applied automatically, but may result in removing “holes” from a polygon. Since this happened in both cases, we reinserted the intended holes by adding slightly smaller, non-intersecting rings for the holes to the polygons.

#### Misplaced polygons

**Fig. 5:**
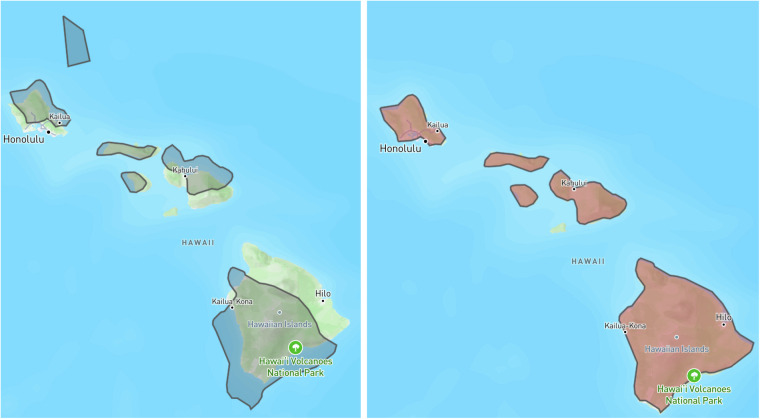
Shapes for the area of the islands of Hawaii overlaid over a geographic map created from OpenStreetmap data using https://geojson.io before and after correction. One “stray” polygon has been removed and the others position-corrected.

Since no information about a CRS was available from the shapefile, we took the coordinates of the shapes to be WGS 84 coordinates – which is the default in most GIS tools. Inspecting the polygons – overlaid on GIS data of the earth’s land masses, islands and reefs from NaturalEarth – revealed that some shapes were incorrectly positioned. Slightly misplaced shapes would be generally acceptable for a dataset of this kind, in line with the characterization of such sets by Bowern 2021^18^. But if misplacement was as severe as to mean that the shape did not intersect with any land mass – but at the same time a correct position was obvious (e.g. because of similar outlines of shape and an island), we corrected the position of the shape. This was done by specifying a point coordinate within a polygon and mapping this to a target coordinate. The translation vector computed from these two points was then added to each point of the polygon specification (see Fig. 5). We corrected the position of 168 polygons, based on priority derived from size of the shape and distance from next land mass.

This method of correcting the position of polygons may seem ad hoc and slightly naive, considering that a likely reason for these misplacements could be a different map projection used when digitizing the data. But the example of the Hawaii islands (see Fig. 5) does not show any systematic deformation or offsets as it would be expected as result of a different (but common) projection, which would affect shapes in roughly the same geographic area in the same way. Our own experience with geo-referencing the Atlas leaves suggests an alternative explanation of the misplacements: If non-linear transformations have been used to geo-reference the leaves and the polygons have been drawn overlaid on these geo-referenced images, then the seeming irregularities we can observe might have been a result of these transformations. In particular areas with few control points may be affected in unexpected ways by non-linear transformations.

#### The Antimeridian problem

**Fig. 6:**
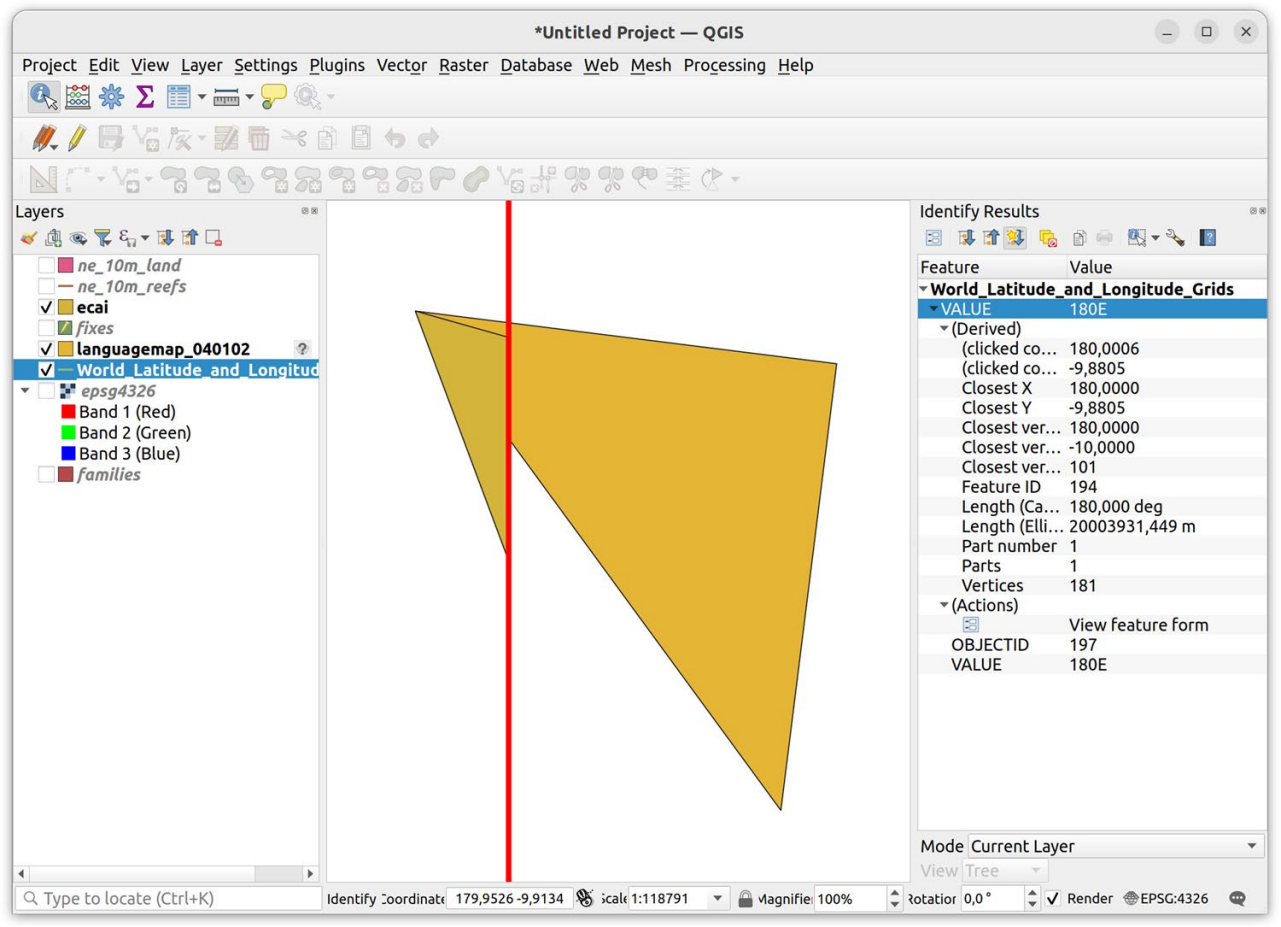
Polygon crossing the antimeridian split in compliance with the GeoJSON specification using the antimeridian package. The algorithm splits each polygon side which crosses the antimeridian in two parts, using the arithmetic mean of the two latitudes as second endpoint. The second polygon of the split geometry is not visible, because it is displayed by QGIS at -180^°^ longitude.

The antimeridian – the line of longitude opposite to the prime meridian through Greenwich – is notorious for causing interoperability problems for geographic mapping tools^35^. Since the antimeridian barely cuts through populated land, it can mostly be ignored when mapping linguistic areas. Languages from the Austronesian family, though, are spoken “on both sides” of the antimeridian and one of the polygons for one shape in the ECAI dataset crosses the antimeridian. The GeoJSON specification^25^ recommends splitting all geometries crossing the antimeridian and this is what we did with the help of the antimeridian package (https://antimeridian.readthedocs.io/en/stable/). We accepted the result of this automated fix despite its obvious imperfection (see Fig. 6), because the original shape (a triangle) did not reflect the proper outline of any of the Tuvaluan islands anyway.

### Aggregating polygons

While we kept the languoid references as fine-grained as possible, matching shapes to the “smallest” linguistic unit that was a valid match in Glottolog (i.e. preferring matching dialects over matching languages), the real requirement for the dataset is to provide “comparable” data – and comparison across languages is typically done on language level, abstracting dialectal differences. Thus, we aggregate all shapes mapped to the same Glottolog **language** (i.e. language-level languoid^27^) and create a single MultiPolygon shape for the speaker area. Since polygons in the shapefile are hand-drawn, they may not actually border, even in cases where they “should”. Thus, to force contiguous areas in these cases, we increase the polygons slightly before merging and then subtract the added buffer from the merged shape using the buffer method implemented in the shapely package^36^.

### CLDF

The Cross Linguistic Data Formats (CLDF)^37^ specify formats to distribute data about languages in an interoperable way. CLDF (v 1.3) has added a *speakerArea* property, which allows transparent linking of geographic information in GeoJSON format to languages in a dataset. Geo-referenced scans can be modeled using CLDF’s *MediaTable* component. Since CLDF also allows adding provenance metadata – which is particularly important for a dataset with quite a history such as this one – it becomes a perfect option as a package format for this dataset.

Making full use of the CLDF eco-system, we also curate the dataset using the cldfbench package^38^, which means:

- We can transparently include “configuration” data, which was not created by the original authors, for example our language-reference table or our polygon corrections
- We can enhance the dataset with functionality via cldfbench custom commands, e.g. to create locally viewable Atlas leaves implemented as HTML page with leaflet

Since the creation of the CLDF dataset is automated, it can be re-run periodically to:

- “Compile” the dataset against new Glottolog versions
- Fix errata, e.g. adding more polygon corrections or correcting language matches

## Data Records

The dataset is available at Zenodo^14^. In the following we describe the set of files and directories making up this dataset. CLDF^37^ is used as package format, linking GeoJSON polygons and scanned Atlas leaves to language (varieties).

### CLDF dataset

As “Language Atlas”, the main object of study in Wurm and Hattori’s Atlas are languages, or more accurately, spoken languages (and language varieties) from the Pacific area. Thus, we format and distribute this dataset according to the CLDF specification to allow transparent access to its content based on Glottocodes, i.e. Glottolog languoid identifiers^26^. These Glottocodes are available from the dataset’s *LanguageTable*, which lists all languoids^39^ for which the dataset provides geographic information. With its focus on languages as the object of study, using CLDF also follows the recommendation of Rantanen *et al*.^13^ to pursue a *data-specific approach* for maximal interoperability.

**Fig. 7:**
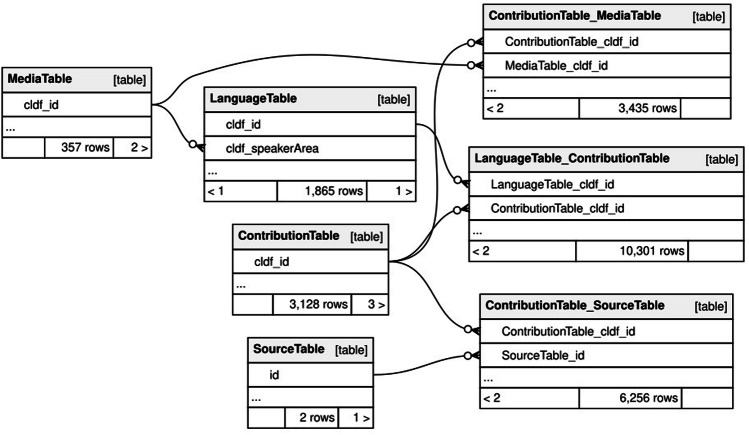
Entity-relationship diagram of the data model of the CLDF dataset.

Using CLDF’s *ContributionTable* component, we list all shape metadata sets from ECAI’s geo-registered dataset and each Atlas leaf as scanned by ECAI as individual contribution in order to be able to keep the provenance-specific metadata. The many-to-many relation between contributions and media files – mediated by the association table *ContributionTable_MediaTable* – links shape metadata to polygons in GeoJSON files and leaf metadata to image files (see Fig. 7).

The dataset’s metadata also contains technical provenance information such as repository versions that the CLDF data is based on, or a list of Python packages (with version numbers) that were used to compile the CLDF dataset. Information about contributors is available as metadata accompanying the released dataset on Zenodo^14^. In line with the metadata provided by ECAI, we list Wurm and Hattori as authors of the data, and ECAI as well as ourselves as editors.

### Polygons

The dataset provides three sets of (Multi)Polygons serialized as GeoJSON *FeatureCollection*s:

1. The corrected shapes from the shapefile with normalized metadata
2. Language level aggregated speaker area MultiPolygons
3. Family level aggregated speaker area MultiPolygons

This GeoJSON data (and metadata) follows the best practice recommendations from Rantanen *et al*.^13^:

**Data type** The speaker area shapes are available as vector data in GeoJSON format.

**Geometry type** The areas are modeled as MultiPolygons or Polygons. File format GeoJSON^25^ is widely used and an open format.

**Coordinate system** GeoJSON uses WGS 84 coordinates

The coordinate reference system for all GeoJSON coordinates is a geographic coordinate reference system, using the World Geodetic System 1984 (WGS 84) datum, with longitude and latitude units of decimal degrees^25^.

**Attribute data** All areas are linked to a Glottolog languoid, and thus additional metadata, using the Glottocode as value of a property *cldf:languageReference*. Through the corresponding metadata in the CLDF ContributionTable, shapes are linked to the ECAI digitization effort and the original Atlas as source.

**Temporal divisioning** Some languages in the Atlas are marked as *extinct* – and often the corresponding Glottolog languoid has the same status. But apart from this, no specific temporal attributes of speaker areas are available.

**Metadata description** The CLDF package links the geographic data to provenance information.

### Geo-referenced Atlas leaves

Using CLDF’s *MediaTable* component (see Fig. 7), we associate each Atlas leaf with a set of media files allowing for reuse and interoperablity with a range of geo-spatial information systems:

image/jpeg Scanned Atlas leaf (in addition reverse side and legend if available) from ECAI

text/plain Ground control points used to geo-reference the scan with QGIS

image/tiff Geo-referenced scan as GeoTIFF for CRS EPSG:4326 - WGS 84

image/jpeg GeoTIFF re-projected to web mercator and translated to JPEG

application/geo+json Bounding box and geo-referenced area of the scan

While the original scans and the ground control points serve mainly as “audit trail” of the processing steps, the GeoTIFF image allows rendering the geo-referenced Atlas leave within GIS systems such as QGIS, and the JPEG in web mercator projection together with its bounding box from the GeoJSON file can be used with common software for interactive web maps, such as the *leaflet* library.

## Technical Validation

To make sure the dataset is usable and useful for research, three aspects of the data must be investigated:

1. How well does the digital data convey the information in the original Atlas?
2. How well does the data match geographical “reality”?
3. How well does the data match linguistic “reality”?

### Relation between the Atlas and its digitization

Without good correspondence between the digitized data and the information in the original Atlas, it would be impossible to transfer the authority of Wurm & Hattori’s work to our dataset. While the Atlas leaves provide richer information than just outlines of speaker areas – e.g. classificatory information – the essential parts are indeed shapes depicting speaker areas for languages and dialects on a geographic map. Thus, the validity of the digitized data can be established by checking the accuracy of location and metadata of the shapes in the shapefile against the scanned Atlas leaves. This checking has been done extensively in the process of “language-referencing” the shapes (see above) and has been facilitated by having all Atlas leaves geo-referenced. The typos spotted in the metadata of the shapefile are the result of this validation.

### Relation between the dataset and the physical world

Many of the languages described in the Atlas are spoken on small islands in the Pacific. Thus, establishing the validity of the data with respect to geographic “reality” comprises investigating whether shapes in the shapefile actually cover islands inhabited by potential speakers. As described above, for several polygons in the original shapefile this was clearly not the case and their position has been manually corrected.

**Fig. 8:**
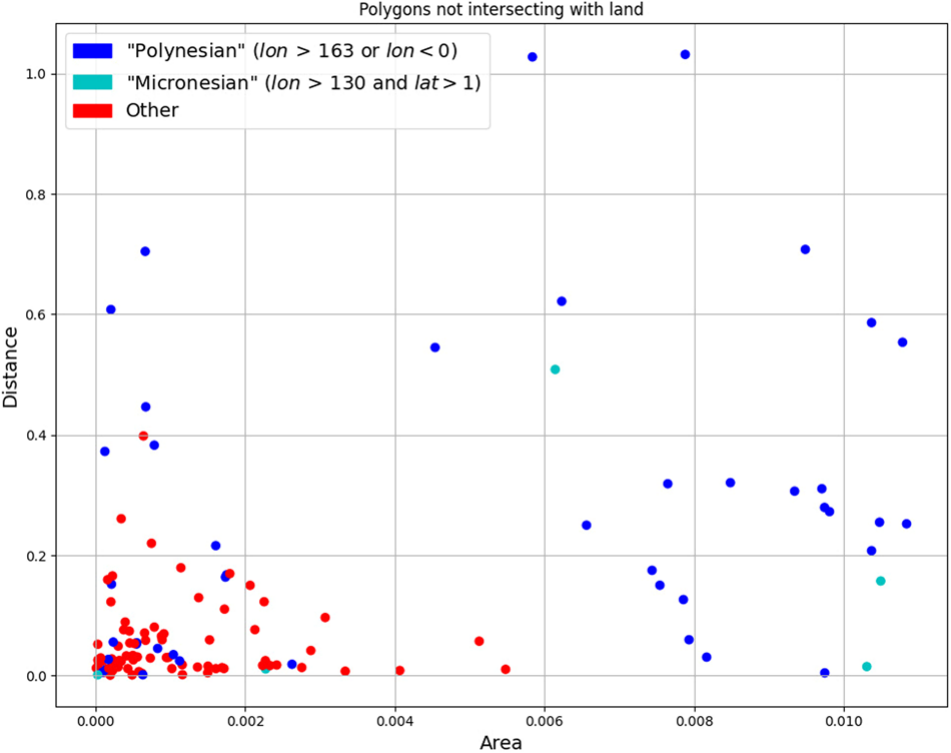
*Area* is computed as cartesian area of the polygon in square degrees. Close to the equator, an area of 1 corresponds roughly to 10, 000 *km*^2^. *Distance* is computed as cartesian distance in degrees. Close to the equator, a distance of 1 corresponds roughly to 100 *km*. For examples of the types of non-intersecting polygons see Figs. 9 and 10.

**Fig. 9:**
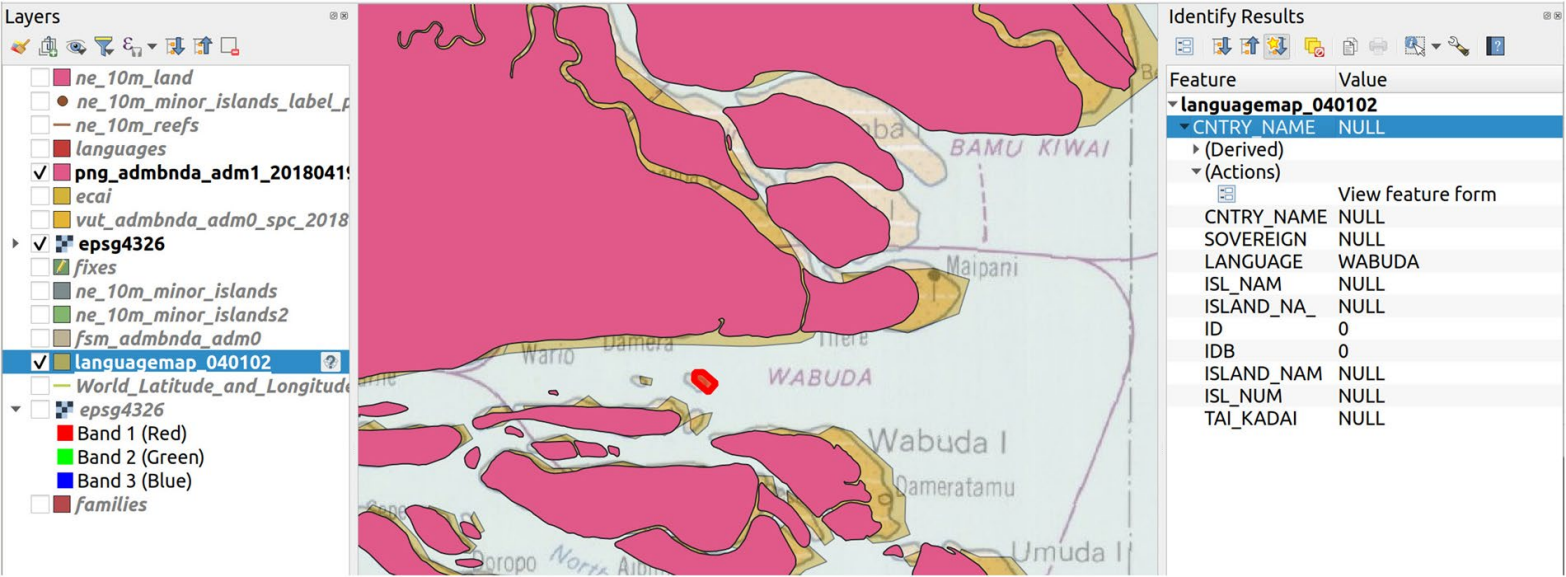
Non-intersecting polygons of the “other” type are typically small shapes, covering coastal islands as shown in this example of the Fly River estuary in Papua New Guinea. The highlighted small island is not even mapped in the PNG administrative boundaries shapefile (pink color).

**Fig. 10:**
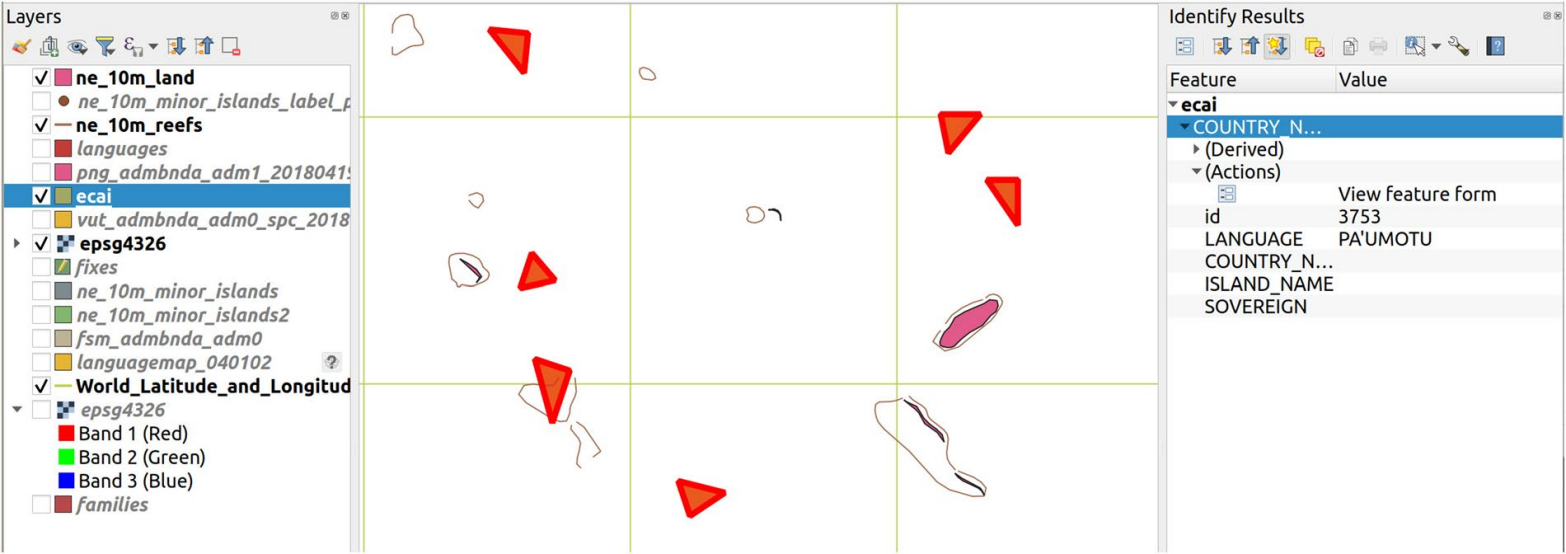
Non-intersecting polygons of the “polynesian” or “micronesian” type are typically small shapes (although large in relation to the land they are supposed to cover) in remote Oceania, surrounded by tiny islands or reefs.

As a proxy for the current state of our knowledge of the world’s physical geography, we take the NaturalEarth large scale data (https://www.naturalearthdata.com/downloads/10m-physical-vectors/). Thus, to validate geographical position of the polygons, we computed the intersection for all polygons in our dataset with any shape of type *Polygon* or *LineString* in the NaturalEarth datasets for land masses, minor islands and reefs. For 145 polygons (which have not been explicitly moved to their position, see above) we found no intersection, suggesting that the polygons do not cover any land. For these polygons we tabulated their area against the distance to the next coast. Plotting this data revealed two categories of such polygons, exhibiting distinctive geographic characteristics (see Fig. 8). Examples of both categories could be explained plausibly (see Figs. 9 and 10). Thus, leaving these polygons in the dataset – without explicitly moving them to some “land” correlates – seemed acceptable. It should be noted that even NaturalEarth’s data on minor islands and reefs does not cover all islands inhabited by humans. For example, the island Mere Lava, on which Merlav is spoken, is not represented in the NaturalEarth data. Its existence, and the position of the polygon for Merlav, could be confirmed though using an administrative boundaries dataset for Vanuatu from the Humanitarian Data Exchange.

The code implementing this validation experiment is available at https://github.com/cldf-datasets/languageatlasofthepacificarea/blob/main/laotpa_commands/landmass_distance.py. The computed table of non-intersecting polygons which was plotted in Fig. 8 is available at https://github.com/cldf-datasets/languageatlasofthepacificarea/blob/main/etc/landmass_distance.csv.

### Relation between the dataset and linguistic “reality”

**Fig. 11:**
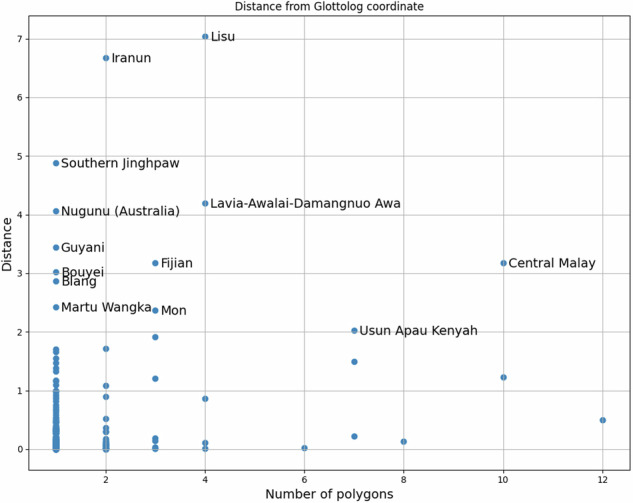
392 language-level shapes with non-zero distance from the corresponding Glottolog centroid coordinate.

To measure how well the dataset corresponds to the current state of our knowledge about the geographic distribution of the world’s languages, we compare it to the geographic information available in Glottolog. Of the 1,769 aggregated MultiPolygons for Glottolog languages 1,765 have a corresponding centroid coordinate in Glottolog 5.0. Of these, 1,286 contained the Glottolog coordinate, corroborating our “language-referencing” as well as the fitness of the dataset for analyzing language data. For another 103 MultiPolygons the Glottolog coordinate was contained in the convex hull of the shape – highlighting one of the advantages of MultiPolygons as geographic correlate for languages over a single point coordinate in the case of languages spoken in different, disconnected areas. For the remaining 376 MultiPolygons we tabulated distances to the Glottolog coordinate and number of polygons (as proxy for “spread” of the language) (see Fig. 11) and examined the outliers, i.e. the 13 languages where the distance from the respective MultiPolygon to Glottolog’s centre-point coordinate was greater than 2 degrees in the cartesian plane, or roughly 200km for regions near the equator (see Table 1). Again, we found all outliers to have plausible explanations.

**Table 1:** Languages in the dataset where the distance of the assigned MultiPolygon from the corresponding Glottolog coordinate is greater than 2 degrees in the cartesian plane or about 200 km near the equator.

Lisu	According to Glottolog, Lisu is spoken in four countries, with the Glottolog coordinate on the border between China and Myanmar, and the Atlas listing areas in Thailand.
Iranum	Iranum is spoken in the Philippines and Malaysia with Glottolog only listing the Malaysian areas, the Atlas listing all areas.
Southern Jinghpaw	Southern Jinghpaw is spoken in China and Myanmar with only a small patch of the area falling within the area mapped in the Atlas.
Lavia-Awalai-Damangnuo Awa	The Glottolog language is listed as being spoken in China, while the Atlas lists small patches in Myanmar.
Nugunu	The Glottolog coordinate has been reported as too far East and will be updated.
Fijian	Relevant areas in the Atlas are mapped to the subgroup Eastern Fijian, because they also include areas where other languages of this subgroup are spoken, leaving just the smaller islands mapped to Fijian.
Guyani	Bowern 2021^18^ assigns roughly the same area to this language as the Atlas.
Central Malay	Glottolog places this language in Kuala Lumpur, but the language is spoken also in Indonesia, Singapore and Thailand, with the Atlas listing areas on Sumatra and Borneo.
Bouyei	Spoken in China and Vietnam with the Glottolog coordinate well inside China and the areas listed in the Atlas within Vietnam.
Blang	Polygon is in a very southern location in Thailand in contrast to the northern location of Glottolog. The location in Thailand represents a recent refugee population and is in this sense not wrong but the Glottolog location is historically and demographically more accurate.
Martu Wangka	The Atlas only lists one dialect of the Glottolog language at roughly the location given in Glottolog for this dialect. But the Glottolog coordinate for the language represents the centre-point for the language.
Mon	The Atlas just lists a couple of very small pockets labeled as MON in Thailand, while Mon is also spoken in Myanmar.
Usun Apau Kenyah	Some areas in the Atlas mapped to this Glottolog language were originally assigned to a supposed language which was subsequently merged into Mainstream Kenyah in ISO 639-3 as well as in Glottolog.

The code computing “Glottolog distances” is available at https://github.com/cldf-datasets/languageatlasofthepacificarea/blob/main/laotpa_commands/glottolog_distance.py. The table of distances (with non-zero distances plotted in Fig. 11) is available at https://github.com/cldf-datasets/languageatlasofthepacificarea/blob/main/etc/glottolog_distance.csv.

The above test fails to validate “language-referencing” on the dialect level in cases where one polygon was mapped correctly and thus contained the Glottolog coordinate but other polygons were aggregated in the language MultiPolygon due to incorrect dialect references. To investigate such situations, we compute the standard deviation of all distances between polygons mapped to the same language. As an example, we can look at polygons in the shapefile for a dialect specified as *Bime, Indonesia*. There are in fact two such polygons, belonging to dialects of *different* languages. If both were mapped to the same language, this would result in a measurement of 3.58 for the above metric.

**Fig. 12:**
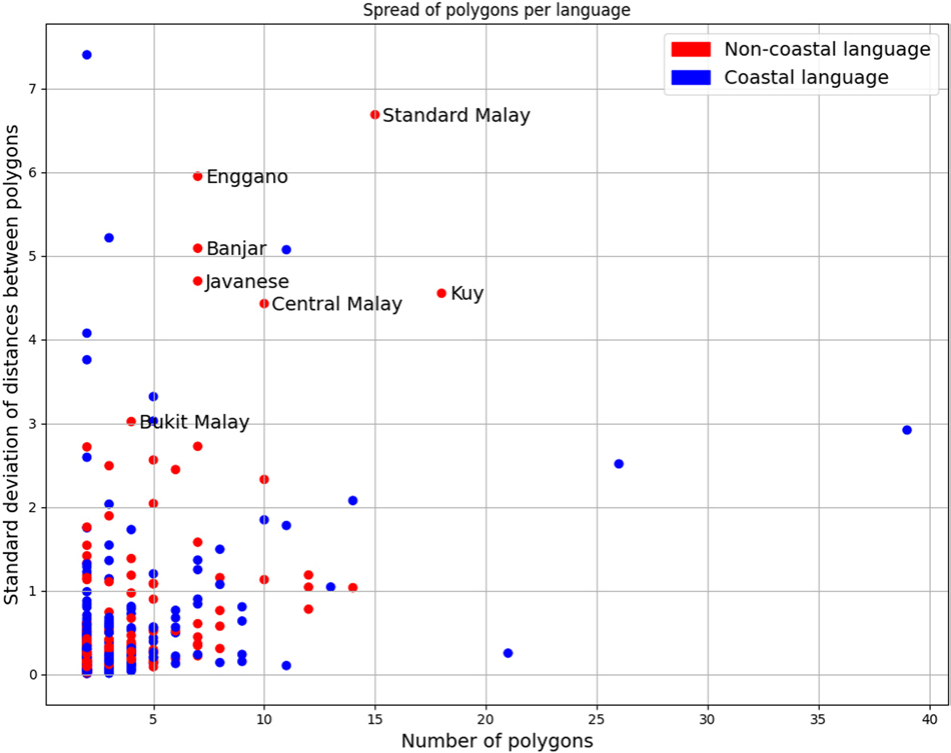
Intra-language polygon spread. Coastal languages spread further. Non-coastal languages with big spread are typically “big”, standardized languages.

The results of this test are shown in Fig. 12. The Bime case from above – if incorrectly mapped – would show up in this plot as labeled red dot. Considering that “migration has always been a major feature of life”^40^ in the Pacific, finding “coastal” languages in addition to “big”, standard languages like Standard Malay as having the biggest spread in the dataset seems very plausible.

The code computing MultiPolygon spread is available at https://github.com/cldf-datasets/languageatlasofthepacificarea/blob/main/laotpa_commands/multipolygon_spread.py. The computed table which served as input for Fig. 12 is available at https://github.com/cldf-datasets/languageatlasofthepacificarea/blob/main/etc/multipolygon_spread.csv.

### Referential integrity

Since the dataset comprises different types of data, stored in different data formats, referential integrity of the dataset as a whole must be guaranteed in order to allow easy reuse. Thus, we made sure the CLDF dataset passes validation – as established by running the cldf validate command as part of the CLDF creation pipeline (https://github.com/cldf-datasets/languageatlasofthepacificarea/blob/main/RELEASING.md). CLDF validation ensures that

- foreign keys between data tables can be resolved,
- data type constraints are enforced,
- features in the GeoJSON files are properly linked to rows in the dataset’s *LanguageTable*.

## Usage Notes

Designed for maximum interoperability, the dataset can be used in a number of ways, with a variety of different tools. General information on using data from CLDF datasets can be found in the CLDF cookbook (https://github.com/cldf/cookbook). In the remainder of this section we will focus on reusing the spatial data.

The polygon data – being available as features in GeoJSON files – can be visualized with tools such as QGIS, but also using web services like https://geojson.io. But GeoJSON data is also easy to use with software like shapely^36^, which supports geometric manipulations of GeoJSON objects such as computing intersections, centroids or distances. It should be noted, though, that often tools (including shapely) support reading spatial data, but perform computations on it simply regarding coordinates as points in the cartesian plane rather than on the surface of the earth. For much of the data of this dataset this is not too much of a problem, considering that close to the equator the WGS 84 CRS behaves almost like the cartesian plane.

If more precision is needed, “proper” geographically informed computations can be done using tools like spatialite – an extension for the SQLite database system. Loading GeoJSON data into a spatialite database can be done with tools like geojson-to-sqlite (https://github.com/simonw/geojson-to-sqlite).

Further details and usage notes – including notes on how to use the geo-referenced scans – are available in the dataset’s repository at https://github.com/cldf-datasets/languageatlasofthepacificarea/USAGE.md.

Since the majority of the information in this dataset has been compiled by humans rather than automated measurement devices, we fully expect it to still contain errors. Thus, detecting errors is part and parcel of using this dataset. But since our pipeline has “maintenance hatches” built-in, we can easily and transparently fix errata and release updated versions of the dataset and will do so periodically. We encourage reporting any issues with the dataset at https://github.com/cldf-datasets/languageatlasofthepacificarea/issues.

## Data Availability

The cldfgeojson^24^ Python package, that was developed and used during the work on this dataset, is released under an Apache 2 license and available from Python’s Package Index and archived on Zenodo. The dataset is curated in a GitHub repository (https://github.com/cldf-datasets/languageatlasofthepacificarea). Released versions are published on Zenodo, accessible via 10.5281/zenodo.11046587. The repository includes the cldfbench code to create the CLDF dataset from the data released by ECAI as well as the code to run the validation experiments described above (https://github.com/cldf-datasets/languageatlasofthepacificarea/blob/main/VALIDATION.md).
